# Prevalence of cervical extension of the thymus in children

**DOI:** 10.1016/j.amsu.2022.103483

**Published:** 2022-03-14

**Authors:** Gonca Koc, Habib Ahmad Esmat, Mehmet Coskun

**Affiliations:** a Department of Pediatric Radiology, Ege University School of Medicine, Izmir, Turkey; b Department of Radiology, Kabul University of Medical Sciences, Kabul, Afghanistan; c Department of Radiology, Dr. Behcet Uz Children's Hospital for Training and Research, Izmir, Turkey

**Keywords:** Thymus, Superior cervical extension, Pediatrics, Ultrasonography

## Abstract

**Background:**

The cervical extension of the thymus is the most common variation. However, this may be mistaken for a soft tissue mass in the neck particularly by the radiologists who are not familiar with the pediatric population and not aware of this variation, leading to unnecessary surgery and increased medical costs. Since the rates of cervicaly extended thymus in children in clinical practice are lacking in Turkey, this study aimed to evaluate the prevalence of cervical extension of the normal thymus in the pediatric population.

**Materials and methods:**

This descriptive cross-sectional study included all pediatric patients who were referred to the radiology department for neck ultrasonography between August–October 2018. A high-frequency probe was implemented and 220 patients (152 male, 68 female) with a mean age of 8.7 ± 4.39 years (ranging from 1 month to 18 years of age) were examined.

**Results:**

Cervical extension of the thymus was detected in 103 patients (46.8%). The age of the patients was found to be significantly lower than the age of the patients whose thymus was not extended (7.87 ± 4.15 years and 9.59 ± 4.46 years, respectively. p = 0.006). The mean craniocaudal length of the thymus that cervically extended was 6.41 ± 2.31 mm. There was no significant difference in the length of the thymus between males, females (6.48 ± 2.12 mm and 6.37 ± 2.46 mm. p = 0.924), and different age groups (p = 0.442).

**Conclusions:**

Approximately half of the children have the cervical extension of the thymus. Thus, radiologists and clinicians should be aware of this entity to avoid unnecessary imaging studies and interventional procedures.

## Highlights

• The cervical extension of the thymus in children is the common anatomic variation.
• It may be mistaken for a soft tissue mass in the neck.
• The clinicians should be aware of this entity to avoid unnecessary imaging and intervention.

## 1 Introduction

The thymus is a primary lymphoid organ that plays an important role in the differentiation of T-cells. It is formed by the fusion of the right and left thymic rudiments, originating from the ectoderm of the third branchial cleft and the endoderm of the third branchial pouch, and moves caudally and medially from the pharyngeal region to the superior anterior mediastinum [1]. Defective pathways of embryologic descent of thymic primordia may lead to a clinical spectrum of anomalies of the thymus [2].

According to our clinical experience of neck ultrasound (US), the cervical extension of the thymus is the most common variation of the thymus. This may be mistaken for a soft tissue mass in the neck particularly by radiologists who are not familiar with the pediatric population and not aware of this variation, potentially leading to unnecessary surgery and increased medical costs [3,4]. To our knowledge, few reports are focusing on the cervical extension of the thymus to reveal the prevalence of this entity [5,6,and7]. Since the rates of cervicaly extended thymus in children in clinical practice are lacking in Turkey, we aimed to evaluate the prevalence of cervical thymic extension in pediatric patients referred to our department for neck ultrasound examination.

## 2 Material and methods

This descriptive cross-sectional, single-centered study was approved by the Institutional Review Board for Ethical Issues in Clinical Research and is compatible with the Declaration of Helsinki. The patients referred to the radiology department for the neck US with various clinical indications between August–October 2018 were included in the study. The informed consent was taken from the parents of the patients. The patients who had any systemic illness, treated for malignancy, or had a history of lower neck or chest surgery, were excluded.

The neck US was performed by two radiologists (G.K., with an 8-years and M. C., with a 2-years of post-residency experience). The scan was performed using a 7–12 MHz high-frequency linear transducer on the Aplio-500 US device (Toshiba Medical System Corporation, Tokyo, Japan). The probe was placed perpendicular in the suprasternal notch in the transverse plane. Without any angulation towards the mediastinum, the cervical extension of the thymus was considered to be ‘positive’, when the thymus-located in the anterior mediastinum abutted the upper border of the manubrium sterni through the neck. The thymus was distinguished with its homogeneous hypoechoic background echogenicity compared to the thyroid with scattered hyperechoic foci resembling a ‘starry sky’. With increasing age, due to physiological fatty replacement, the echogenicity of the cervically extended thymus could increase.

The length of the extended thymus, from the upper margin of the manubrium to the superior edge of the thymic tissue on the transverse plane was measured in millimeters and recorded. The images containing the most upper extent of the thymus at midline or near the left or right of the midline were used for measurement.

Patients were grouped according to sex and age intervals (0–1, 2–5, 6–10, 11–15, >15-year-old) and compared statistically in terms of the length of the cervically extended thymus.

Statistical analysis of the data was performed in SPSS, Version 25 program (IBM, Armonk, New York). Pearson Chi-Square Test was used in comparing categorical data between groups. Due to the lack of normal distribution of continuous data, Kruskal Wallis H was implemented for comparisons between more than two groups and Mann Whitney U statistical analysis for comparisons between two groups. p<0.05 was considered statistically significant.

This work has been reported in line with the STROCSS 2021 criteria [8].

## 3 Results

**Fig. 1:**
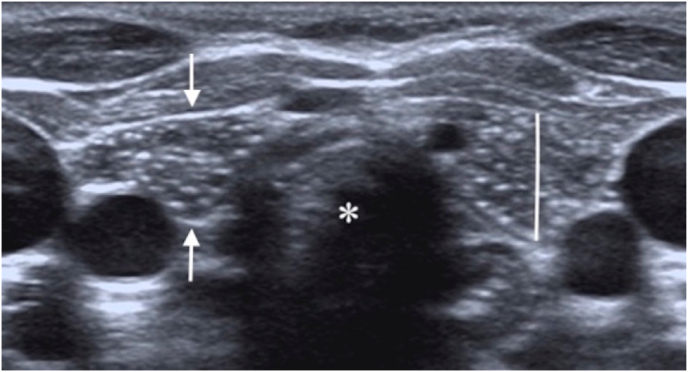

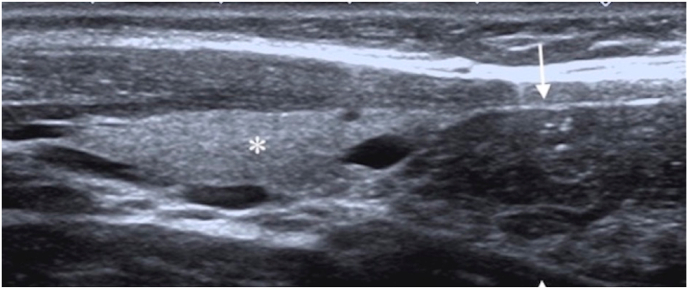
a. The ultrasonography image acquired on the transverse plane reveals cervical extension of the thymus (arrows) located anterior to the cervical trachea (asterisk) in a one-year-old boy. The thymic length was measured on the same plane in mm. Note the typical ‘starry sky’ appearance of normal thymus tissue. Fig. 1b. On the longitudinal plane, the cervically extended thymus tissue (arrows) is just below the thyroid lobe (asterisk).

220 patients (152 male, 68 female; ranging from 1 month to 18 years of age) with a mean age of 8.7 ± 4.39 years were included in the study. 103 patients (46.8%) had cervically extended thymus. The location of the extended thymus was in the midline and inferior portion of the neck not exceeding the thyroid lobes (Fig. 1). The age of the patients was found to be statistically significantly lower than the age of the patients whose thymus was not extended (7.87 ± 4.15 years and 9.59 ± 4.46 years, respectively. p = 0.006). The mean length of the thymus above the manubrium was 6.41 ± 2.31 mm. There was no significant difference in the length of the thymus between males and females (6.48 ± 2.12 mm and 6.37 ± 2.46 mm, respectively. p = 0.548). The length of the extended cervical thymus by age intervals is illustrated in Table 1. Statistically significant difference was not seen among the age groups (X^2^ = 3.743, p = 0.442).

**Table 1:** The distribution of mean length of the cervically extended thymus by age groups.

		Length (mm)		X^2^	p
Age (years)	n	Length	Median (Min.-Max.)		
0–1	7	8,16 ± 3,39	7,2 (3,4–13,2)	3743	0,442
2–5	29	6,42 ± 2,2	6,1 (3,3–11,9)		
6–10	43	6,48 ± 1,94	6,1 (3–11,6)		
11–15	23	5,8 ± 2,57	6 (0–12,5)		
15	5	6,24 ± 2,77	6,1 (3,3–10,1)		

In 4 patients (1, 8%, the thymus tissue was observed to be ectopically located on the neck independent of the mediastinum thymus. The ectopic thymus was most frequently detected inferiorly to both thyroid lobes.

## 4 Discussion

The current study revealed the prevalence of cervical extension of the thymus as 46.8% in a pediatric population using US examination. The study paints a complete picture of the presence and frequency of the cervical thymus in children, which is consistent with the concept that cervical thymus is a frequent and normal anatomic finding. Furthermore, our study may be one of the few crass sectionally designed US studies in the literature investigating the presence of the thymus with cervical extension in children.

The thymus is the central lymphoid organ of infancy and is responsible for T-cell differentiation. Thymus arises bilaterally from the 3rd and to a lesser extent 4th branchial pouches, starting from the fifth or sixth gestational weeks. During the seventh week, the thymic primordia located in the pharynx starts its descent medially and caudally. In the eighth week, thymic tissue reaches the anterior mediastinum and left and right primordia fuse at their lower poles [9]. The defects in thymus migration may cause the thymus to become ectopically located, either partly or totally, as seen mostly in the cervical region. The extension of the thymus to the inferior border of the thyroid gland is frequently observed in routine neck US examinations, especially during the inspiratory phase [10]. However, the cervical extension of the thymus may be mistaken for a soft tissue mass in the neck, particularly in children and young adults, potentially leading to unnecessary surgery and increased medical costs [3,4].

The ultrasound is the first-line modality of choice for the evaluation of superficial head and neck structures without the use of ionizing radiation, iodinated contrast material, sedation, and/or anesthesia. This modality can provide quick and cost-effective acquisition of the information, including the location, size, shape, internal contents (solid or cystic), and vascularity of any mass, as well as its relationship to nearby anatomic structures [11].With the development of the technology on ultrasound resolution, more detailed examinations that are revealing findings either previously unreported or even unsuspected are possible [12]. The presence of a pathological lesion in the neck in children always arouses diagnostic alertness. Moreover, encountering a suspicious neck lesion in a child generates significant stress for the patient and the parents, who need to know promptly whether the lesion may be malignant [13].

In the literature, there is a limited number of studies focusing on the cervical extension of the thymus. In a study reviewing PET/CT examinations acquired in children and young adults with malignancy and under the age of 20 years for 2 months, the superior thymic extension was reported as 11% [6]. However, in another study by Costa et al, the frequency of cervical extension ot the thymus among the patients scanned with MRI for various clinical indications but not for malignancy was reported as 66.5% [5]. In the retrospectively designed study by Qin Yong et al, comprising a relatively largest series to date and evaluating MRI features as diagnostic criteria, the incidence of superior cervical extension of the thymus was reported to be 72.0% in children with a decreasing frequency with age [7]. Our study was compatible with the literature, especially conducted in the pediatric population who does not have any sign of malignancy, revealing that thymus should be expected to extend cervically at least in half of the pediatric age group.

The thymus has a unique US appearance, a homogeneous hypoechoic background echogenicity, compared to the thyroid, with scattered hyperechoic foci resembling ‘starry sky’ due to the mixture of lymphoid tissue and fat. Continuity with the thymus located in the anterior mediastinum and the US characteristics resembling normal thymus tissue is the features helping establish the diagnosis of 'cervically extended thymus' [14].

The cervical thymic extension above the manubrium in the midline should not be confused with a pathologic condition. Criteria for differentiating the normal finding of cervical thymic extension from pathologic mass is that cervical extension appears as a soft tissue lobe, extending above the upper margin of the manubrium anterior to the trachea and great vessels with direct vicinity of the mediastinal thymic tissue and should not deform or compress vessels or airway [5,15]. In our study, the typical sonographic features of thymus tissue and the same criteria for diagnosis were taken into consideration.

**Fig. 2:**
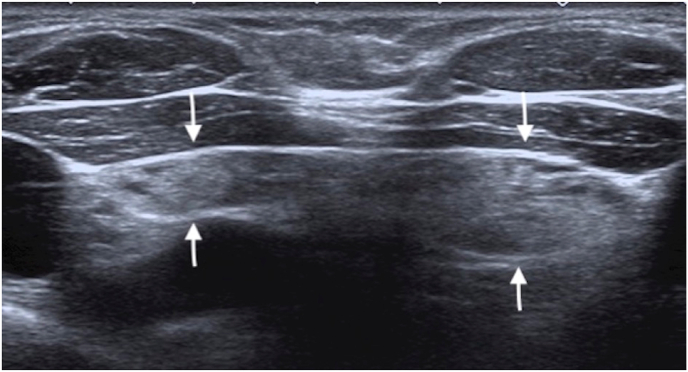
The ultrasonography image shows the fatty replacement of the thymus (arrows) in a 10-year-old girl.

In the current study, we demonstrated no significant difference between the sex and age groups in terms of frequency of cervical extension. However, the patients without cervically extended thymus were significantly older than those in whom cervical extension was detected. The increased echogenicity of the thymus due to increasing fatty replacement with the age may interfere with discrimination of the thymus from the fatty tissue of the neck (Fig. 2).

There were several limitations in this study. Although this study, to our knowledge, is one of the few studies implementing the US to determine the prevalence of superior cervical extension of the thymus in children, the study had a relatively small study population. Future US studies with a larger sample size would emphasize the entity of the cervical thymic extension. Since the echogenicity of the thymus increases with age due to fatty replacement, discrimination of the cervically extended thymus from surrounding adipous tissue may be challenging. More than half of the pediatric patients were older than ten years of age and this might have been interfering with the detection of cervical extension of the thymus.

## 5 Conclusion

The findings of our study shows that approximately 50% of the children have a cervical extension of the thymus. The extended thymus tissue is often located in the midline at the suprasternal notch level in the neck and continued with the mediastinal thymus, having the typical thymus echogenicity. However, with increasing age due to fatty replacement the thymus echogenicity increases and cervical extension may not be detected on US examination. Thus, radiologists and clinicians should be aware of the high frequency of cervical extension of the thymus as a variation in pediatric patients to avoid unnecessary imaging studies and interventional procedures.

## Provenance and peer review

Not commissioned, externally peer reviewed.

## Declaration of conflict of interest

The authors declare no conflict of interest.
