# Supplementary material for: Prevalence of cervical extension of the thymus in children
Source: Ann Med Surg (Lond). 2022 Mar 14;76:103483. doi: 10.1016/j.amsu.2022.103483 (PMC8940946; doi:10.1016/j.amsu.2022.103483)
Supplement: Multimedia component 1 [file mmc1.docx]

| The STROCSS 2019 Guideline | | |
| --- | --- | --- |
| Item no. | **Item description** | **Page** |
| TITLE | | |
| 1 | **Prevalence of cervical extension of the thymus in children-A prospective study** |  |
| ABSTRACT | | |
| 2a | - Introduction: The cervical extension of the thymus is the most common variation of the thymus. However, this may be mistaken for a soft tissue mass in the neck, particularly by radiologists who are not familiar with the pediatric population and not aware of this variation, potentially leading to unnecessary surgery and increased medical costs. Since the rates of cervicaly extended thymus in children in clinical practice are lacking in Turkey, this study aimed to evaluate the prevalence of cervical extension of the normal thymus in the pediatric population. |  |
| 2b | - Methods: This prospective, observational study included all pediatric patients who were referred to the radiology department for neck ultrasonography between August-October 2018. A high-frequency probe was implemented and 220 patients (152 male, 68 female) with a mean age of 8.7 ± 4.39 years (ranging from 1 month to 18 years of age) were examined. |  |
| 2c | - Results: Cervical extension of the thymus was detected in 103 patients (46.8%). The age of the patients was found to be statistically significantly lower than the age of the patients whose thymus was not extended (7.87 ± 4.15 years and 9.59 ± 4.46 years, respectively. P= 0.006). The mean craniocaudal length of the thymus that cervically extended was 6.41 ± 2.31 mm. There was no significant difference in the length of the thymus between males, females (6.48 ± 2.12 mm and 6.37 ± 2.46 mm. P= 0.924), and different age groups (p = 0.442). |  |
| 2d | - Conclusion: Approximately half of the children have the cervical extension of the thymus. Thus, radiologists and clinicians should be aware of this entity to avoid unnecessary imaging studies and interventional procedures. |  |
| INTRODUCTION | | |
| 3 | Introduction: The thymus is a primary lymphoid organ that plays an important role in the differentiation of T-cell. It is formed by the fusion of the right and left thymic rudiments, originating from the ectoderm of the third branchial cleft and the endoderm of the third branchial pouch, and moves caudally and medially from the pharyngeal region to the superior anterior mediastinum [1]. Defective pathways of embryologic descent of thymic primordia may lead to a clinical spectrum of anomalies of the thymus [2].  According to our clinical experience of neck ultrasound (US), the cervical extension of the thymus is the most common variation of the thymus. This may be mistaken for a soft tissue mass in the neck, particularly by radiologists who are not familiar with the pediatric population and not aware of this variation, potentially leading to unnecessary surgery and increased medical costs [3, 4]. To our knowledge, few reports are focusing on the cervical extension of the thymus particularly no US study to reveal the prevalence of this entity [5, 6, and 7]. Since the rates of cervicaly extended thymus in children in clinical practice are lacking in Turkey, we aimed to evaluate the prevalence of cervical thymic extension in pediatric patients referred to our department for neck ultrasound examination. |  |
| METHODS | | |
| 4a | Registration and ethics  This prospective study was approved by the Institutional Review Board for Ethical Issues in Clinical Research and is compatible with the Declaration of Helsinki. |  |
| 4b | Ethical Approval: This prospective study was approved by the Institutional Review Board for Ethical Issues in Clinical Research and is compatible with the Declaration of Helsinki. |  |
| 4c | Not applicable |  |
| 4d | Patient Involvement in Research   - The patients referred to the radiology department for the neck US with various clinical indications between August-October 2018 were included in the study. |  |
| 5a | Study Design: A prospective, single -centred) |  |
| 5b | - Setting: The patients referred to the radiology department of EGE university teaching hospital for the neck US with various clinical indications between August-October 2018 were included in the study. |  |
| 5c | Cohort Groups: Not applicable |  |
| 5d | Subgroup Analysis: Not applicable |  |
| 6a | - Participants: The patients referred to the radiology department for the neck US with various clinical indications between August-October 2018 were included in the study. The patients who had any systemic illness, treated for malignancy, or had any history of lower neck or chest surgery, were excluded |  |
| 6b | - Recruitment: The patients referred to the radiology department for the neck US with various clinical indications between August-October 2018 were included in the study. The patients who had any systemic illness, treated for malignancy, or had any history of lower neck or chest surgery, were excluded |  |
| 6c | - Sample Size: 220 patients (152 male, 68 female) |  |
| INTERVENTION AND CONSIDERATIONS | | |
| 7a | Pre-intervention Considerations: Not applicable |  |
| 7b | Intervention: Not applicable |  |
| 7c | Intra-Intervention Considerations: Not applicable |  |
| 7d | Operator Details: The neck US was performed by two radiologists (G.K., with an 8-years and M. C., with a 2-years of post-residency experience). The scan was performed using a 7-12 MHz high-frequency linear transducer on the Aplio-500 US device (Toshiba Medical System Corporation, Tokyo, Japan). The probe was placed perpendicular in the suprasternal notch in the transverse plane. Without any angulation towards the mediastinum, the cervical extension of the thymus was considered to be ‘positive’, when the thymus-located in the anterior mediastinum abutted the upper border of the manubrium sterni through the neck. The thymus was distinguished with its homogeneous hypoechoic background echogenicity, compared to the thyroid with scattered hyperechoic foci resembling a ‘starry sky’. With increasing age, due to physiological fatty replacement, the echogenicity of the cervically extended thymus could increase.  The length of the extended thymus, from the upper margin of the manubrium to the superior edge of the thymic tissue on the transverse plane was measured in millimeters and recorded. The images containing the most upper extent of the thymus at midline or near the left or right of the midline were used for measurement. |  |
| 7e | Quality Control:   - The neck US was performed by two radiologists (G.K., with an 8-years and M. C., with a 2-years of post-residency experience) to reduce variation |  |
| 7f | Post-Intervention Considerations: Not applicable |  |
| 8 | Outcomes: Not applicable |  |
| 9 | Statistics: the following areas are described comprehensively   - Statistical tests, packages/software used, and interpretation of significance - Confounders and their control, if known - Analysis approach (e.g. intention to treat/per protocol) - Sub-group analysis, if any |  |
| RESULTS | | |
| 10a | - Participants: 220 patients (152 male, 68 female) with a mean age of 8.7 ± 4.39 years (ranging from 1 month to 18 years of age) were examined. Cervical extension of the thymus was detected in 103 patients (46.8%). The age of the patients was found to be statistically significantly lower than the age of the patients whose thymus was not extended (7.87 ± 4.15 years and 9.59 ± 4.46 years, respectively. P= 0.006). The mean craniocaudal length of the thymus that cervically extended was 6.41 ± 2.31 mm. There was no significant difference in the length of the thymus between males, females (6.48 ± 2.12 mm and 6.37 ± 2.46 mm. P= 0.924), and different age groups (p = 0.442). |  |
| 10b | - Participant Comparison: The age of the patients was found to be statistically significantly lower than the age of the patients whose thymus was not extended (7.87 ± 4.15 years and 9.59 ± 4.46 years, respectively. P= 0.006). The mean craniocaudal length of the thymus that cervically extended was 6.41 ± 2.31 mm. There was no significant difference in the length of the thymus between males, females (6.48 ± 2.12 mm and 6.37 ± 2.46 mm. P= 0.924), and different age groups (p = 0.442). |  |
| 10c | Intervention: Not applicable |  |
| 11a | - Outcomes: Approximately half of the children have the cervical extension of the thymus. Thus, radiologists and clinicians should be aware of this entity to avoid unnecessary imaging studies and interventional procedures. |  |
| 11b | Tolerance: Not applicable |  |
| 11c | Complications: Not applicable |  |
| 12 | Key Results: Cervical extension of the thymus was detected in 103 patients (46.8%). The age of the patients was found to be statistically significantly lower than the age of the patients whose thymus was not extended (7.87 ± 4.15 years and 9.59 ± 4.46 years, respectively. P= 0.006). The mean craniocaudal length of the thymus that cervically extended was 6.41 ± 2.31 mm. There was no significant difference in the length of the thymus between males, females (6.48 ± 2.12 mm and 6.37 ± 2.46 mm. P= 0.924), and different age groups (p = 0.442).the following areas are described comprehensively |  |
| DISCUSSION | | |
| 13 | Discussion:   - The current study revealed the prevalence of cervical extension of the thymus as 46.8% in a pediatric population by US examination. The study paints a complete picture of the presence and frequency of the cervical thymus in children, which is consistent with the concept that cervical thymus is a frequent and normal anatomic finding. Furthermore, our study may be one of the few prospectively designed US studies in the literature investigating the presence of the thymus with cervical extension in children.   In the literature, there is a limited number of studies focusing on the cervical extension of the thymus. In a study reviewing PET/CT examinations acquired in children and young adults with malignancy and under the age of 20 years for 2 months, the superior thymic extension was reported as 11% [6]. However, in another study by Costa et al, the frequency of cervical extension among the patients scanned with MRI for various clinical indications but not for malignancy was reported as 66.5% [5]. In the retrospectively designed study by Qin Yong et al, comprising a relatively largest series to date and evaluating MRI features as diagnostic criteria, the incidence of superior cervical extension of the thymus was reported to be 72.0% in children, with a decreasing frequency with age [7]. Our study was compatible with the literature, especially conducted in the pediatric population who does not have any sign of malignancy, thymus should be expected to extend cervically in the pediatric age group at least in half of the patients. |  |
| 14 | Strengths and Limitations:  There were several limitations in this study. Although this study, to our knowledge, is one of the few studies implementing the US to determine the prevalence of superior cervical extension of the thymus in children, the study had a relatively small study population. Future prospective US studies with a larger sample size would emphasize the entity of the cervical thymic extension. Since the echogenicity of the thymus increases with age due to fatty replacement, discrimination of the cervically extended thymus from surrounding adipous tissue may be challenging. More than half of the pediatric patients were older than ten years of age and this might have been interfering with the detection of cervical extension of the thymus. |  |
| 15 | Implications and Relevance:  Although this study, to our knowledge, is one of the few studies implementing the US to determine the prevalence of superior cervical extension of the thymus in children, the study had a relatively small study population. Future prospective US studies with a larger sample size would emphasize the entity of the cervical thymic extension. |  |
| CONCLUSION | | |
| 16 | Conclusions:   - Approximately 50% of the children have a cervical extension of the thymus. - With increasing age, due to fatty replacement the thymus echogenicity increases and cervical extension may not be detected on US examination. - Radiologists and clinicians should be aware of the high frequency of cervical extension of the thymus as a variation in pediatric patients to avoid unnecessary imaging studies and interventional procedures. |  |
| DECLARATIONS | | |
| 17a | Conflicts of interest:  The authors declare no conflict of interest. |  |
| 17b | Funding:  No financial support was provided for this study. |  |
